# Texture synthesis for generating realistic-looking bronchoscopic videos

**DOI:** 10.1007/s11548-023-02874-6

**Published:** 2023-05-10

**Authors:** Lu Guo, Werner Nahm

**Affiliations:** Karlsruhe Institute of Technology, Kaiserstraße 12, Karlsruhe, 76131 Germany

**Keywords:** Texture synthesis, GANs, Endoscopy, Bronchoscopy, Synthetic data generation, Video augmentation

## Abstract

****Purpose**:**

Synthetic realistic-looking bronchoscopic videos are needed to develop and evaluate depth estimation methods as part of investigating vision-based bronchoscopic navigation system. To generate these synthetic videos under the circumstance where access to real bronchoscopic images/image sequences is limited, we need to create various realistic-looking image textures of the airway inner surface with large size using a small number of real bronchoscopic image texture patches.

****Methods**:**

A generative adversarial networks-based method is applied to create realistic-looking textures of the airway inner surface by learning from a limited number of small texture patches from real bronchoscopic images. By applying a purely convolutional architecture without any fully connected layers, this method allows the production of textures with arbitrary size.

****Results**:**

Authentic image textures of airway inner surface are created. An example of the synthesized textures and two frames of the thereby generated bronchoscopic video are shown. The necessity and sufficiency of the generated textures as image features for further depth estimation methods are demonstrated.

****Conclusions**:**

The method can generate textures of the airway inner surface that meet the requirements for the texture itself and for the thereby generated bronchoscopic videos, including “realistic-looking,” “long-term temporal consistency,” “sufficient image features for depth estimation,” and “large size and variety of synthesized textures.” Besides, it also shows advantages with respect to the easy accessibility to required data source. A further validation of this approach is planned by utilizing the realistic-looking bronchoscopic videos with textures generated by this method as training and test data for some depth estimation networks.

## Introduction

Vision-based bronchoscopic navigation system, as an alternative to the electromagnetic navigation system [1], helps to localize the bronchoscope during the endobronchial inspection and diagnostic procedures by applying video-CT registration techniques [2]. Motivated by its advantages of low cost, less impact from tissue deformations, and no requirement for additional equipment setup [3], it has been the focus of research in bronchoscopic navigation in recent years. There, several 2D–3D registration approaches have been proposed, among which the approaches trying to recover the geometrical structure of the airway based on depth estimation from monocular bronchoscopic video images prove to be more robust to illumination variations [4]. Some examples of such depth estimation approaches include conventional “Structure from Motion” (SfM) method, and learning-based depth estimation methods utilizing different networks architectures. For further development and evaluation of these methods (especially learning-based methods due to their well-proven outstanding performance for monocular depth estimation), a large amount of bronchoscopic videos and a known ground truth of depth maps corresponding to the bronchoscopic video images are required.

**Fig. 1:**
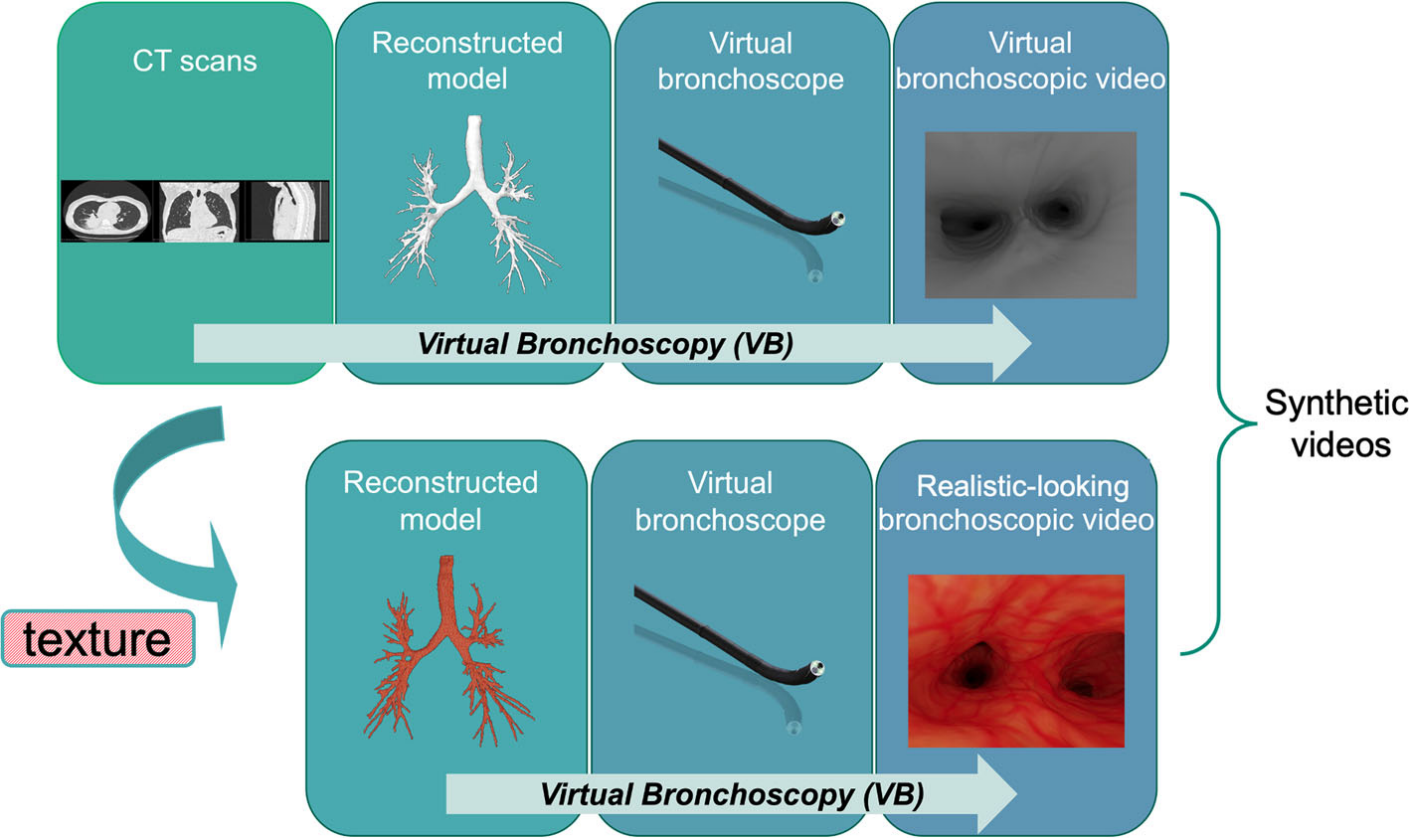
Overview of texture mapping and virtual bronchoscopy process

However, the access to real intra-operative bronchoscopic videos is sometimes limited, and it is difficult to obtain the ground-truth data. One way to overcome this obstacle is to use synthetic videos with their rendered depth ground truths. Virtual bronchoscopy, as a non-invasive technology allowing the creation of bronchoscope-like inner views of human airway, has been used to generate large datasets of synthetic bronchoscopic video images, along with their corresponding rendered depth maps [5]. Here, the videos are recorded by a virtual bronchoscope whose camera and light parameters are selected based on the setup of common real bronchoscopes. It flies along a pre-defined pathway through the airway models, which are reconstructed from patients’ CT scans, while the illuminations are configured to meet the inverse square law. However, if the airway models are not colored and textured as they are in the real case (and in this case, we call the captured synthetic videos “virtual videos”), the domain gap between virtual and real videos will lead to a performance drop when transferring the depth estimation models trained on these virtual data to real scenes [6].

To narrow the gap, it is necessary to either transfer the virtual videos to realistic-looking ones, or directly color and texture the airway models to make the captured videos look real. Except for “realistic-looking,” two additional requirements are proposed for the generated realistic-looking videos: (1) The videos should be long-term temporally consistent. (2) The task-related features, which in our case are the vascular patterns that are important for the motion detection step in the depth estimation task, should be created or preserved and sufficient for the depth estimation. To achieve this goal, one option is to transfer virtual bronchoscopic videos to realistic-looking ones by extending generative adversarial networks-based (GANs-based) unpaired image translation to “virtual to real” domain video translation. A related example can be found in [7]. It is also possible to combine the unpaired translation with neural rendering [8], as is done in work [9]. However, these approaches call for a large amount of real bronchoscopic videos/image sequences as training data, which are not always easy to obtain. Another option is to directly texture the whole airway models and capture the realistic-looking bronchoscopic videos by performing the virtual bronchoscopy. An overview of this process is shown in Fig. 1. (Note that the “virtual videos/images” and “realistic-looking video/images” are grouped according to whether the video/images have realistic-looking colors and textures, and they are both “synthetic videos/images” compared to “real video/images.”) The key part of this work is the generation of textures used for the texture mapping.

**Fig. 2:**
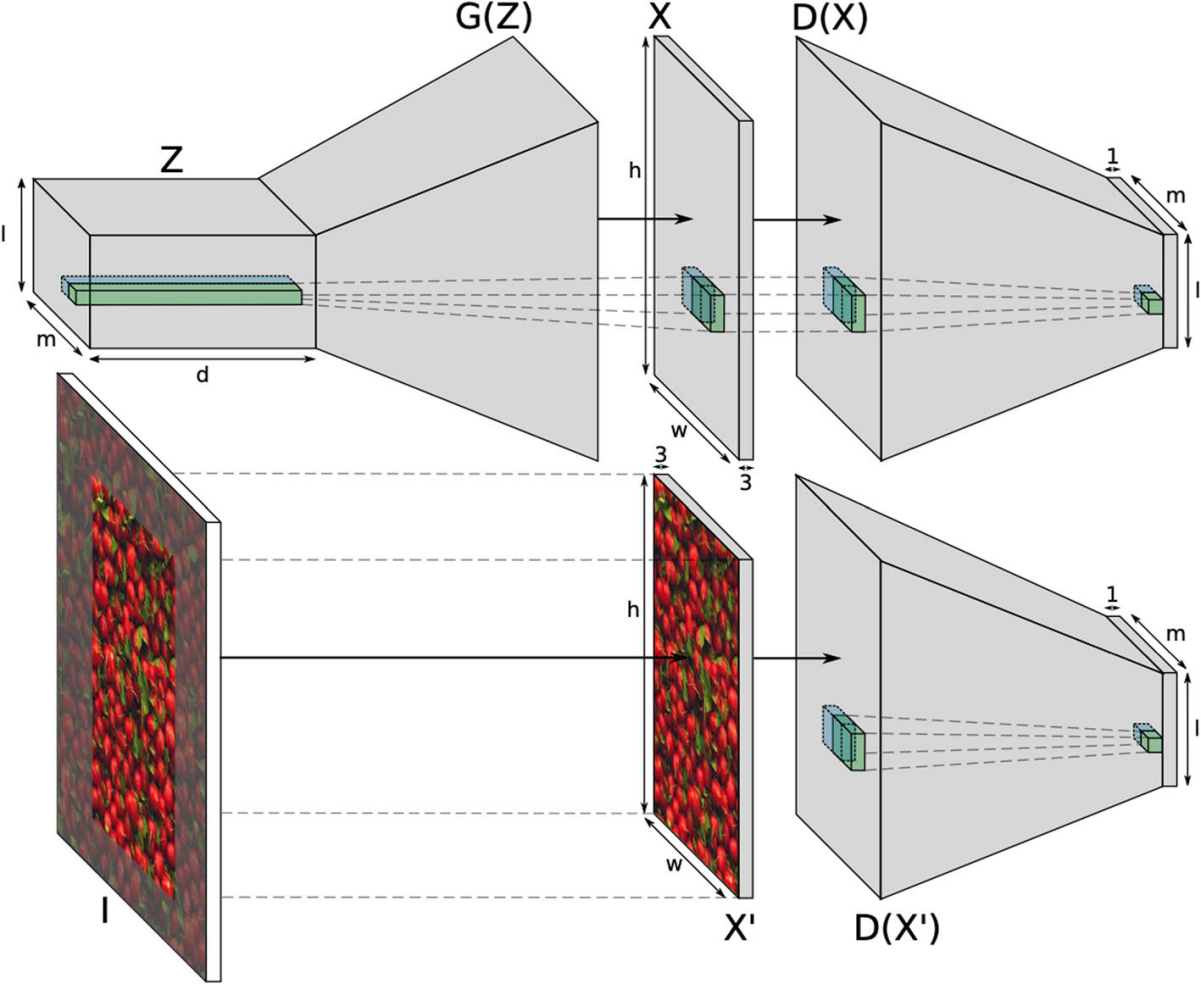
Spatial GAN model (SGAN) from [12]

In our case, we proposed three requirements for the generated textures: (1) The textures should mimic the textures of the human airway inner surface since the captured synthetic bronchoscopic videos should be realistic-looking. (2) The textures should be large enough to cover the whole airway models, and the repetition of patterns in neighboring areas should be avoided (for the purpose of proper feature matching and motion detection which are depth estimation-related). (3) The generated textures should be various for obtaining diverse bronchoscopic videos. On the other hand, as sources for generating the textures, we only have a limited number of real bronchoscopic images with a small size (not larger than $500 \times 500$ pixels). To meet the requirements based on our sources, we can make use of procedural vessel-like textures, for example, the random Voronoi textures, or create realistic-looking image textures. Since the reality of the procedural textures is deficient with regard to a bronchoscopic scene, as shown in Fig. 5a, it is advantageous either to utilize existing large realistic-looking image textures, which are in most cases unavailable, or to create such textures from our sources. In this work, we synthesize image textures learning from a small number of real bronchoscopic image patches containing airway inner surface textures, with the aim of generating realistic-looking bronchoscopic videos.

## Methods

In recent years, several data-driven texture synthesis approaches have been proposed. Compared to the traditional texture synthesis methods by non-parametric sampling [10] or by matching statistical descriptors of images [11], data-driven methods allow to learn texture models from input images and effectively produce textures of arbitrary size. Considering that our need is to generate image textures of large size mimicking human airway inner surface textures from a small number of real bronchoscopic image patches of small size (not larger than $256 \times 256$ pixels), in this work, we chose to apply the spatial generative adversarial networks (SGAN) introduced by Jetchev et al. [12] to synthesize textures of the airway inner surface.

The SGAN is an extension of the well-known generative adversarial networks (GANs). The GANs are often used to generate realistic-looking images of such high quality that they can even fool humans by learning a generator network which captures the data distribution and a discriminator network which tries to distinguish between the generated and training data simultaneously [13]. In SGAN [12], the generator *G*(*z*) maps a randomly sampled spatial array $Z \in {\mathbb {R}}^{l\times m\times d}$ from a prior distribution $p_Z(Z)$ into an RGB image $X \in {\mathbb {R}}^{h \times w\times 3}$ via a stack of fractionally strided convolutional layers. Here, the *l*, *m* are the spatial dimensions and the *d* is the number of channels, while the *h* and *w* represent the height and width of the generated image *X*, respectively. Each slice of *Z* is restricted at position $\lambda$ and $\mu$, where $\lambda , \mu \in {\mathbb {N}}$ with $1 \le \lambda \le l$ and $1 \le \mu \le m$. Similarly, the discriminator *D* shares a symmetric architecture of the generator *G*, consisting also a stack of convolutional layers. It takes either a generated image *X* or an image patch $X' \in {\mathbb {R}}^{h \times w\times 3}$ of an image *I* from the training dataset as input and gives out a 2D field of size $l\times m$ containing probabilities that indicate whether the input is real or generated. The image patch $X'$ is extracted at a random position of image *I* which defines the true data distribution $p_{data}$. Besides, the architecture of both generator and discriminator is modified and adapted from the DCGAN architecture [14]. They both apply purely convolutional architecture without any fully connected layers which makes the manipulation of the spatial dimensions (*l* and *m*) without changing the weights possible. And that enables the generation of textures with arbitrary size. A schematic diagram of the SGAN model is shown in Fig. 2.

**Fig. 3:**
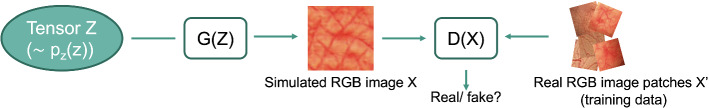
Spatial GAN in our case

**Fig. 4:**
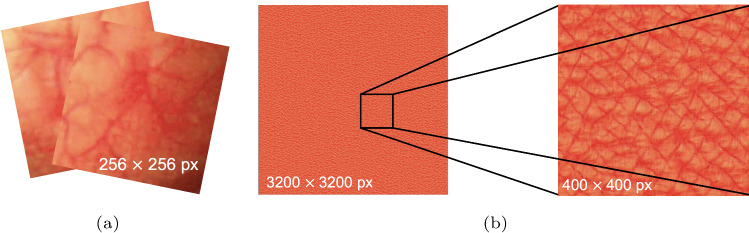
**a** An example of training data of size $256 \times 256$ pixels; **b** an example of synthesized textures using SGAN of size $3200 \times 3200$ pixels, and a close-up of a $400 \times 400$ pixels’ patch of it

**Fig. 5:**
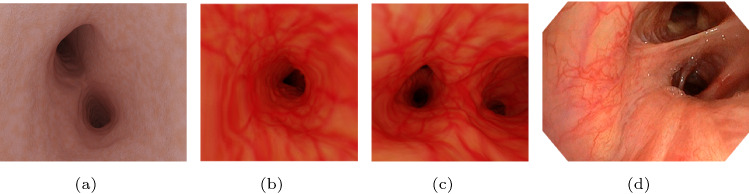
**a** A frame from a generated bronchoscopic video with random Voronoi textures; **b**, **c** two frames from a realistic-looking bronchoscopic video with the texture shown in Fig. 4**b**; **d** a frame from a real bronchoscopic video

The discriminator and the generator are optimized simultaneously over all spatial dimensions as:

1 $$\begin{aligned} \begin{aligned} \min _G\max _D V(D,G)&= \frac{1}{lm}\sum ^l_{\lambda =1}\sum ^m_{\mu =1}{\mathbb {E}}_Z\sim p_Z(Z)\\&\times [\textrm{log}(1-D_{\lambda \mu }(G(Z)))]\\&+\frac{1}{lm}\sum ^l_{\lambda =1}\sum ^m_{\mu =1}{\mathbb {E}}_X'\sim p_{\textrm{data}}(X')\\&\times [\textrm{log} D_{\lambda \mu }(X')] \end{aligned} \text { [12]}\nonumber \\ \end{aligned}$$

In the original work, SGAN has been successfully applied to generate textures learning from one single image and to composite textures from multiple images. It has been tested using natural textures of radishes, flowers, satellite images, etc. [12]. In our case, as shown in Fig. 3, the true data distribution is defined by a small number of texture image patches cut from real bronchoscopic images containing vessel-like textures. To create various textures of the airway inner surface, we took different numbers/combinations of these texture image patches as training data for the SGAN. The synthesized textures were mapped onto the airway models, and the realistic-looking videos were then recorded by performing the virtual bronchoscopy, as mentioned in Sect. 1.

## Results

As an example, we train the SGAN with two texture image patches of size $256 \times 256$ pixels from different real bronchoscopic images, as shown in Fig. 4a. Figure 4b shows one of the synthesized textures of the airway inner surface with the size of $3200 \times 3200$ pixels. More details can be found in a $400 \times 400$ pixels subset of the created texture (see Fig. 4b).

After applying the texture mapping and virtual bronchoscopy process using the synthesized texture shown above, we get a simulated realistic-looking bronchoscopic video that is long-term temporal consistent since the scenes, including the textures, are static and unchanged during the video recording. Two frames of the video are shown in Fig. 5b, c. Compared to the video frame which is generated by texturing the airway model with random Voronoi textures (see Fig. 5a), we see the scenes with texture produced by SGAN better mimic the real bronchoscopic scene (for instance, Fig. 5c), especially with respect to the vascular patterns.

**Fig. 6:**
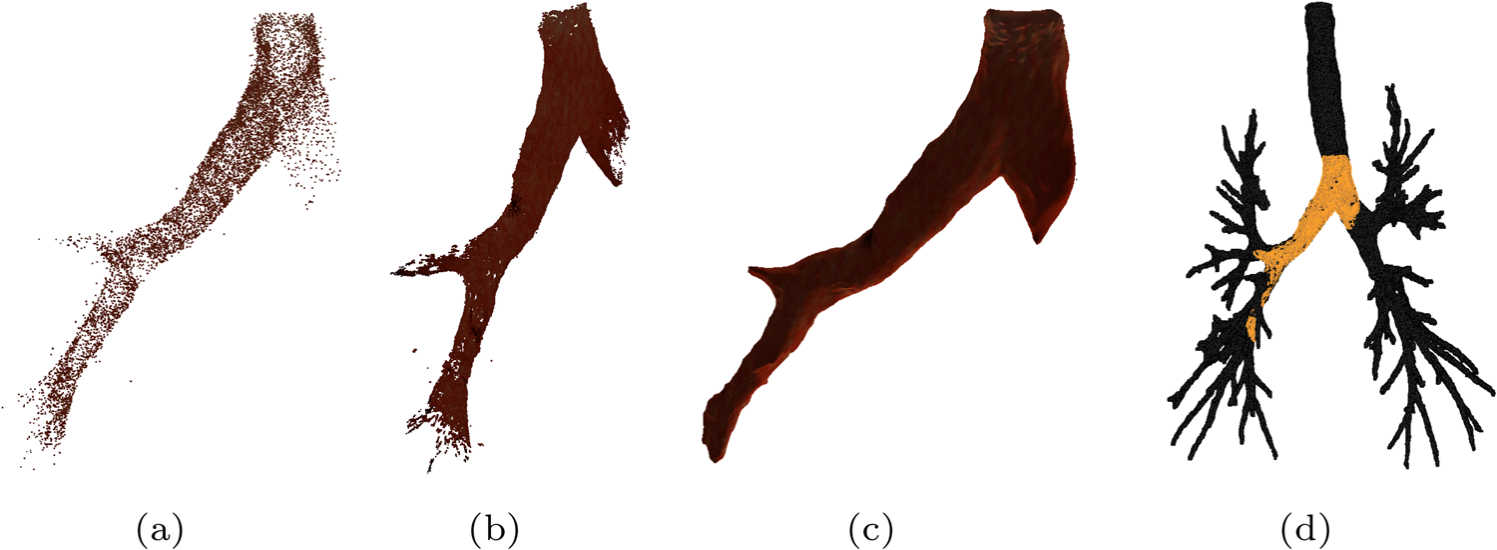
Reconstructed results from sampled 91 images with our generated texture shown in Fig. 4**b** using *Agisoft Metashape*; **a** reconstructed sparse point cloud; **b** reconstructed dense point cloud; **c** reconstructed model; **d** registration of the reconstruction (yellow) to the segmented airway model from CT scans (dark)

**Table 1:** Performance of *Agisoft Metashape* on sampled realistic-looking and virtual images in stage of point cloud reconstruction

Input image type	Number of input images	Number of images in aligning result of SfM	Number of cloud points (sparse)
Sampled realistic-looking images	91	91	9230
Sampled virtual images	91	26	691

Additionally, in order to figure out whether the generated textures are sufficient for the targeted downstream task, i.e., the monocular depth estimation from bronchoscopic video images, an experiment has been carried out. A sequence of 91 images is sampled from the realistic-looking bronchoscopic video with the texture shown in Fig. 4b. And a popular commercial SfM-based software *Agisoft Metashape* is used to reconstruct a 3D model from these images since SfM-based approaches estimate depth and rely heavily on features in the images, which in our case are offered mainly by the textures. That makes it suitable to evaluate texture-related performances. *Agisoft Metashape* aligns the input images and calibrates the camera using the SIFT technique and bundle block adjustment, creates sparse and dense point cloud (see Fig. 6a, b, respectively), and builds a mesh (Fig. 6c) on this basis [15]. Here, depth information corresponding to each image is also calculated and is crucial to the proper 3D reconstruction. In this approach, since textures play a role as features for the feature matching across the images, which will impact the number of aligned images and the number of generated sparse cloud points, we observe these two values as hints of whether our generated textures are sufficient considering further application in developing and evaluating depth estimation method.

Table 1 shows the results that from the 91 realistic-looking images with our generated texture, *Agisoft Metashape* is capable of aligning all the input images and reconstructing a sparse point cloud containing more than 9000 points. That means our generated texture can offer enough features for the SfM-based depth estimation method. At the same time, the reasonable reconstructed results reported in Fig. 6a, b and c also prove the sufficiency of our generated texture when it is applied to further task of depth estimation. This is further revealed by a valid registration of the reconstructed model to the segmented airway model from the corresponding patient’s CT scans shown in Fig. 6d, with an RMSE of 3.5882 mm, using ICP registration.

On the other hand, whether it is necessary to generate such textures can be answered by feeding the software *Agisoft Metashape* with 91 virtual bronchoscopic images with no textures in them, which exactly correspond to the 91 realistic-looking images mentioned above. The results in Table 1 give a clear positive conclusion with the small number of aligned images and reconstructed sparse cloud points. It is also supported by the fact that no dense point cloud can be reconstructed from the virtual images in the experiment.

## Conclusion

In this work, we have applied a GANs-based texture synthesis method SGAN to create image textures of the airway inner surface learning from a limited number of small real bronchoscopic texture patches. The requirement of the data source is much easier to meet compared to other approaches. It enables the generation of realistic-looking bronchoscopic videos when large real bronchoscopic image sequences datasets or large realistic-looking image textures are unavailable. To our knowledge, this is the first trial to generate image textures for airway models with the mentioned purpose based on GANs.

When we evaluate our synthesized textures based on the requirements for the texture itself and for the generated bronchoscopic videos (see Sect. 1), we can see:

1. Realistic-looking: The idea behind the GANs is that the generator is trained in such a manner that the discriminator can hardly distinguish the generated data from the desired ones. In our case, it means that the generator should produce image textures as authentic as possible compared to the textures in the real bronchoscopic images. It theoretically supports the characteristic of “realistic-looking” of our synthesized textures. In addition, a common way to measure quality of texture synthesis is to visually evaluate the generated samples [12]. As is also done in several related works [12, 16], a professional has verified the reality of the generated bronchoscopic videos with the created textures by a visual assessment.
2. Long-term temporal consistency: The process of obtaining realistic-looking bronchoscopic videos by applying texture mapping and virtual bronchoscopy guarantees the long-term temporal consistency of the generated videos, since the scenes and textures remain unchanged during the video recording.
3. Sufficient image features for depth estimation: The necessity and sufficiency of our generated textures as image features for further development and evaluation of monocular depth estimation methods applying to bronchoscopic video images have been proved. It is done by testing the feature-related performance in 3D reconstruction from our realistic-looking bronchoscopic images using a commercial SfM-based software *Agisoft Metashape*, and registering this reconstruction to the segmented airway model from the corresponding patient’s CT scans.
4. Large size of synthesized textures: Since the image textures should be large enough to avoid the repetition of patterns in neighboring areas in the realistic-looking bronchoscopic videos, the SGAN is advantageous, thanks to its ability to produce textures of arbitrary size from smaller texture patches.
5. Variety of synthesized textures: The SGAN can produce various textures when fed with different training data from one single to multiple texture patches. The variety of these produced textures are important to mitigate the risk of the further depth estimation method being too specific to the given data.

However, we must note that our approach still has shortcomings. For example, the artifacts, such as the specular highlights, which are commonly seen in a real bronchoscopic scene, and significantly impact depth estimation from bronchoscopic video images, cannot be reconstructed using our approach. An alternative to address this limitation is to use the GANs-based unpaired video translation method and the neural rendering mentioned in Sect. 1. However, this requires a large amount of real bronchoscopic videos.

In future, we plan to further prove the effectiveness of the approach described in this work by utilizing the generated realistic-looking bronchoscopic videos (together with the corresponding ground truth of depth maps) as training and test data for some depth estimation networks. Meanwhile, we can also see potential applicability of this work in other applications, such as texture synthesis for other kinds of medical images, cancer screening, and endoscopic simulation systems for surgical training.

## Data Availability

Data used in this publication were generated by the National Cancer Institute Clinical Proteomic Tumor Analysis Consortium (CPTAC) [17, 18].
